# Current Understanding of the Innate Control of Toll-like Receptors in Response to SARS-CoV-2 Infection

**DOI:** 10.3390/v13112132

**Published:** 2021-10-22

**Authors:** Hi Eun Jung, Heung Kyu Lee

**Affiliations:** Graduate School of Medical Science and Engineering, Korea Advanced Institute of Science and Technology (KAIST), Daejeon 34141, Korea; euphoric@kaist.ac.kr

**Keywords:** SARS-CoV-2, COVID-19, Toll-like receptor (TLR), cytokine storm, hyperinflammation

## Abstract

The global coronavirus disease 2019 (COVID-19) pandemic, caused by severe acute respiratory syndrome-coronavirus-2 (SARS-CoV-2) infection, threatens the entire world. It has affected every aspect of life and increased the burden on both healthcare and socioeconomic systems. Current studies have revealed that excessive inflammatory immune responses are responsible for the severity of COVID-19, which suggests that anti-inflammatory drugs may be promising therapeutic treatments. However, there are currently a limited number of approved therapeutics for COVID-19. Toll-like receptors (TLRs), which recognize microbial components derived from invading pathogens, are involved in both the initiation of innate responses against SARS-CoV-2 infection and the hyperinflammatory phenotype of COVID-19. In this review, we provide current knowledge on the pivotal role of TLRs in immune responses against SARS-CoV-2 infection and demonstrate the potential effectiveness of TLR-targeting drugs on the control of hyperinflammation in patients with COVID-19.

## 1. Introduction

The coronavirus disease 2019 (COVID-19) pandemic remains a threat to human life. Since the first case of infection was reported in China in December 2019, severe acute respiratory syndrome-coronavirus-2 (SARS-CoV-2), which causes COVID-19, has been rapidly transmitted from person to person worldwide. Confirmed cases of global COVID-19 have surpassed 238 million, and more than 4.85 million people have died from the disease according to researchers at Johns Hopkins University. Patients with COVID-19 have reported a wide range of symptoms, ranging from mild to severe illness, with several studies suggesting that robust expression of proinflammatory cytokines is involved in the pathogenesis of the most severe cases [1,2,3]. The dysregulated release of cytokines, including tumor necrosis factor (TNF)-α, interferon (IFN)-γ, interleukin (IL)-1β, and IL-6, are related to a poor prognosis in patients with COVID-19 [4,5,6,7,8].

SARS-CoV-2, which belongs to the *Betacoronavirus* genus, is an enveloped, positive-sense, single-stranded RNA (ssRNA) virus. In March 2020, the World Health Organization declared the outbreak of COVID-19 a pandemic. Before the outbreak, two members of the *Betacoronavirus* genus, SARS-CoV and Middle East respiratory syndrome-coronavirus (MERS-CoV), had previously been documented to have caused epidemics: SARS-CoV infected 8422 people and killed 916 in 2003 according to the WHO, and MERS-CoV caused 2574 confirmed cases with 886 deaths from 2012 until June 2021 [9,10]. These zoonotic coronaviruses circulated in bats, jumping to humans via intermediate hosts, and resulted in public health emergencies. Compared with these two epidemics, the current COVID-19 outbreak has led to an unprecedented burden on both healthcare and socioeconomic systems.

While the mechanism by which SARS-CoV-2 triggers immune responses has not been fully elucidated, much research has been devoted to investigating the virological characteristics of SARS-CoV-2 and host immune responses. In this review, we summarize the current knowledge of the mechanisms in which host Toll-like receptors (TLRs) recognize SARS-CoV-2.

## 2. Virological Features of SARS-CoV-2

*Betacoronavirus* is one of four genera (*Alphacoronavirus*, *Betacoronavirus*, *Deltacoronavirus*, and *Gammacoronavirus*) of the Coronavirinae subfamily, which belongs to Coronaviridae family. Among these, *Alphacoronavirus* (HCoV-229E and HCoV-NL63) and *Betacoronavirus* (HCoV-OC43, HCoV-HKU1, SARS-CoV, MERS-CoV, and SARS-CoV-2) can infect humans. Prior to the emergence of SARS-CoV, human coronaviruses were most commonly responsible for the common cold [11] and mild upper respiratory tract infections. However, highly pathogenic coronaviruses (SARS-CoV, MERS-CoV, and SARS-CoV-2) have emerged in human populations, resulting in serious health problems and even death.

The intact SARS-CoV-2 virion is surrounded by a lipid envelope that contains the envelope protein (E), membrane protein (M), and spike glycoprotein (S). The genome of SARS-CoV-2 consists of large, single-stranded positive RNA (from 29.8 to 29.9 kb) that contains 14 open-reading frames (ORFs) encoding 27 proteins [12,13,14,15]. The genome sequence of SARS-CoV-2 displays 79.0% homology with SARS-CoV and 51.8% with MERS-CoV [14]. Nucleocapsid (N) proteins form complexes with genomic RNA for genome packaging [16,17].

During viral entry into host cells, the surface trimeric S glycoprotein mediates receptor recognition and viral-host cell membrane fusion. The host protease furin cleaves the S protein into S1 and S2 subunits for preactivation, and the receptor-binding domain (RBD) of S1 binds to angiotensin-converting enzyme 2 (ACE2) expressed on the surfaces of host cells [18,19,20,21]. Then, SARS-CoV-2 enters host cells by either direct fusion [22,23] or endocytosis [24,25]. The transmembrane protease serine subtype 2 (TMPRSS2) on host cells leads to a conformational change in the S protein by cleaving the S2′ site to initiate membrane fusion [25,26]; additionally, the endosomal cysteine proteases cathepsins B and L promote the fusion of viral and endosomal membranes [25,27,28]. Following viral entry into host cells, the viral RNA genome is released into the host cell cytoplasm and is translated into the viral proteins required for viral replication. 

**Figure 1:**
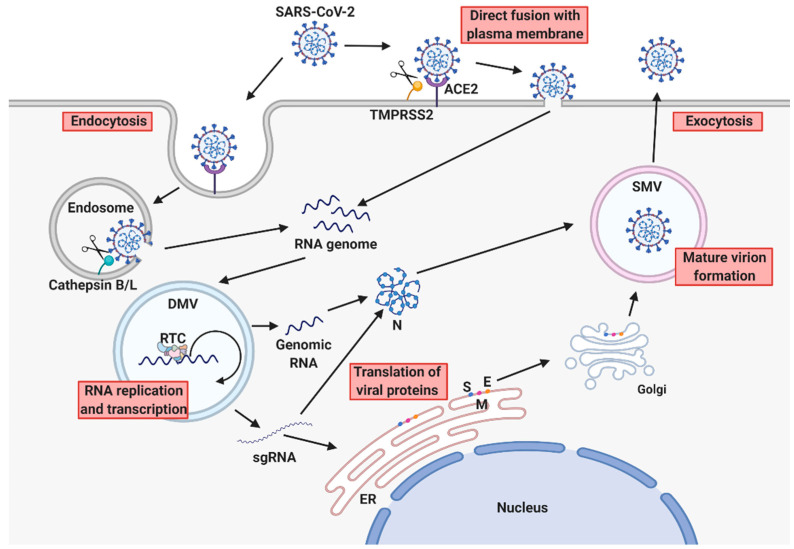
SARS-CoV-2 replication cycle. Viral entry of SARS-CoV-2 is initiated by the recognition of the host cell receptor ACE2 via the RBD of the S glycoprotein. After binding to host cell receptors, SARS-CoV-2 enters cells by endocytosis or direct fusion with the plasma membrane. The host proteases TMPRSS2 and cathepsins B and L mediate the proteolytic cleavage of the S protein, triggering membrane fusion and viral genome release into the cytoplasm. The RTC carries out viral RNA synthesis in DMVs, and newly produced viral RNAs and proteins are delivered to SMVs for assembly of new viruses. Finally, virions are secreted by exocytosis. This figure was created by BioRender.com accessed on 10 September 2021 (BioRender, Toronto, ON, Canada).

SARS-CoV-2 replicates in the host cell cytoplasm [29]. Initially, viral polymerase proteins are directly translated from the RNA genome, which the polymerases use as a template [30]. Two major ORFs, ORF1a and ORF1b, encode pp1a and pp1b polyproteins that are proteolytically cleaved into 16 nonstructural proteins (nsps) [15,31]. The nsps compose the viral replication and transcription complex (RTC), and nsp12, the RNA-dependent RNA polymerase, synthesizes viral RNAs, including genomic RNA and subgenomic (sg) RNA, in double-membrane vesicles (DMVs) in the perinuclear region [29,32,33,34]. The other ORFs encode structural proteins S, E, M, and N, as well as accessory proteins [31]. sgRNAs are translated into viral proteins, and newly synthesized viral RNAs and proteins are translocated to single-membrane vesicles (SMVs) where viral assembly occurs, with new virions released from infected cells by exocytosis [33,35] (Figure 1).

## 3. TLRs Are Involved in SARS-CoV-2 Recognition

During an infection, the immune system works to protect the host from foreign invaders. Innate immune responses are the first line of defense against pathogens entering the body and are responsible for the priming of adaptive immune responses. Innate immune cells express pattern-recognition receptors, such as TLRs, retinoic acid-inducible gene-I-like receptors, nucleotide-binding oligomerization domain-like receptors, C-type lectin receptors, and absent in melanoma-2-like receptors, to recognize pathogen-associated molecular patterns (PAMPs) on pathogens [36]. Among them, TLRs play a crucial role in the activation of innate immune responses against various pathogens (Table 1). TLRs are expressed in immune cells, fibroblasts, and epithelial cells, including type II pneumocytes which highly express ACE2 in the airways [37,38,39]. The activation of TLRs initiates the recruitment of adaptor molecules, such as MyD88 and TRIF, that lead to the subsequent production of type I IFNs and inflammatory cytokines via the activation of nuclear factor-κB (NF-κB) and IFN-regulatory factors (IRFs).

**Figure 2:**
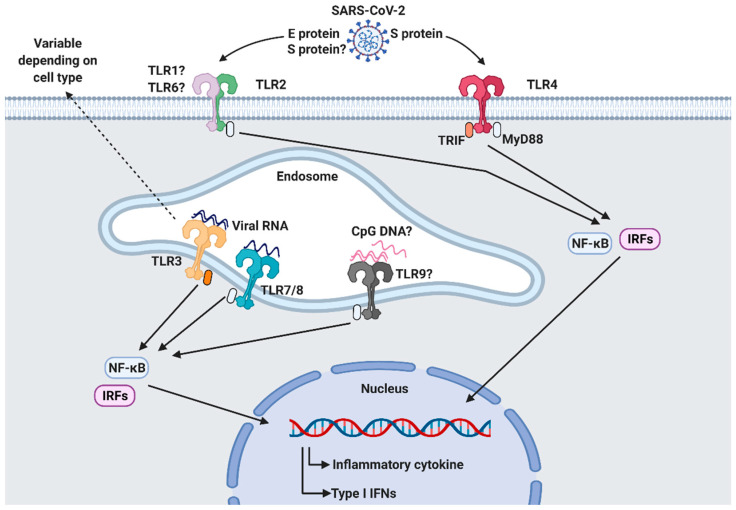
SARS-CoV-2 recognition by Toll-like receptors (TLRs). TLRs are responsible for recognizing pathogen-associated molecular patterns (PAMPs) derived from invading pathogens. Surface TLR2 and TLR4 and intracellular TLR3, TLR7/8, and TLR9 are thought to be involved in the sensing of SARS-CoV-2 infection. Activated TLRs initiate downstream signaling pathways by recruiting adaptor molecules, such as MyD88 and TRIF, which results in the subsequent production of inflammatory cytokines and type I IFNs through transcription factors NF-κβ and IRFs. This figure was created by BioRender.com accessed on 10 September 2021 (BioRender, Toronto, ON, Canada).

According to the latest research [40,41,42,43,44,45], several TLRs are involved in the sensing of PAMPs from SARS-CoV-2. It has been suggested that TLR2, TLR3, TLR4, TLR7/8, and TLR9 contribute to antiviral responses against SARS-CoV-2 infection (Figure 2).

### 3.1. Cell Surface TLRs

Currently, 10 members of the TLR family have been identified in humans. TLRs are currently classified into two categories based on their cellular localization [64]: TLR1, TLR2, TLR4, TLR5, TLR6, and TLR10 belong to cell surface TLRs that recognize microbial components including proteins and lipids derived from invading pathogens, whereas TLR3, TLR7, TLR8, and TLR9 are intracellular TLRs that sense nucleic acid ligands [65].

TLR2 is a surface receptor that recognizes diverse ligands derived from viruses, bacteria, fungi, and parasites [66]. TLR2 forms heterodimers with TLR1 and TLR6, utilizing MyD88 for signal transduction. While the involvement of TLR2 in immune responses against coronavirus infections has not been elucidated, a recent study revealed that the SARS-CoV-2 E protein is sensed by TLR2 [40,67]. Zheng and colleagues reanalyzed the expression of MyD88 and TLRs in patients with different severity grades of COVID-19 using a public dataset and found that the expression of MyD88, TLR1, TLR2, TLR4, TLR5, TLR8, and TLR9 was increased in patients with severe to critical illness. To clarify which TLRs were essential for the sensing of *Betacoronavirus*, they infected bone marrow-derived macrophages deficient in TLR2, TLR4, TLR7, or TLR9 with mouse hepatitis virus, which belongs to the *Betacoronavirus* genus, and found that TLR2 deficiency resulted in the abrogated expression of inflammatory cytokine genes. They then investigated the role of TLR2 in SARS-CoV-2 infection using human peripheral blood mononuclear cells treated with a TLR2 inhibitor. They performed experiments using heat-inactivated SARS-CoV-2 to identify the viral components responsible for TLR2 activation, and structural proteins were identified as promising targets. Among four viral structural proteins identified, the E protein activated the TLR2 signaling pathway. In addition, the authors found that the SARS-CoV-2 E protein induced TLR2-dependent inflammation in mice and that the TLR2 inhibitor protected mice from lethal SARS-CoV-2 infection, indicating that the SARS-CoV-2 E protein is a novel ligand for TLR2 activation.

However, a separate group identified the SARS-CoV-2 S protein as a TLR2 ligand in non-peer reviewed preprints [68]. They observed that recombinant S protein induced inflammatory mediators in macrophages, monocytes, and human lung epithelial A549 cells via the activation of the TLR2-mediated NF-κB pathway. Indeed, intraperitoneal injection of recombinant S protein triggered TLR2-mediated proinflammatory cytokine production in mice. While further studies are required, these results offer valuable insight into TLR2-dependent immune responses against SARS-CoV-2 infection.

TLR4 is well known to recognize lipopolysaccharides produced by Gram-negative bacteria. Early in the COVID-19 pandemic, an in silico study suggested a possible interaction between the SARS-CoV-2 S protein and TLR4 [69]. Investigators used a computational approach to study the interaction between the S protein and host receptors. Interestingly, they found that the SARS-CoV-2 S protein strongly bound to TLR4, suggesting a potential role for TLR4 in SARS-CoV-2 recognition. This hypothesis has been supported by other evidence demonstrating that TLR4 and its downstream signaling molecules are significantly upregulated in patients with severe COVID-19 compared to those with mild illness [40,70]. Recently, two groups confirmed that the S protein leads to proinflammatory cytokine production in monocytes and macrophages in a TLR4-dependent manner [43,44]. However, the identity of TLR4-binding sites in the S protein remains unclear. Shirato and Kizaki showed that the S1 subunit (residues 16–671) induced the activation of the NF-κB and mitogen-activated protein kinase pathways as well as subsequent proinflammatory cytokine production in macrophages [44]. S1-induced proinflammatory responses in macrophages were abrogated following treatment with a TLR4 antagonist or by transfection with TLR4 siRNA. On the other hand, Zhao and colleagues demonstrated that only the trimeric S protein, rather than its N-terminal domain (NTD) (residues 1–307) or RBD (residues 319–541), activates immune responses in macrophages [43]. They suggested that a conformational binding site composed of the RBD and NTD of the S protein was likely to interact with TLR4. Further studies are required to elucidate the relationship between TLR4 and the S protein.

### 3.2. Intracellular TLRs

Nucleic acid-sensing TLRs (TLR3, TLR7, TLR8, and TLR9) are localized in endosomes to prevent the recognition of self-DNA or -RNA. As SARS-CoV-2 is an ssRNA virus and produces double-stranded RNA (dsRNA) during replication in host cells [34,71], intracellular RNA sensors are thought to be involved in the recognition of SARS-CoV-2 infection. TLR3 senses dsRNA in endosomes, and TLR3 stimulation leads to the production of proinflammatory cytokines and type I IFNs via the activation of the TRIF signaling pathway. In contrast, ssRNA is recognized by TLR7 and TLR8, which use MyD88 as a downstream adapter protein. Recently, the roles of TLR3 and TLR7 in antiviral responses following SARS-CoV-2 infection were identified using three-dimensional lung multicellular spheroids [41]. The relative expression levels of TLR3 and TLR7, as well as the production of proinflammatory cytokines and type I IFNs, were elevated in SARS-CoV-2-infected multicellular spheroids, and both IRF3 and NF-κB appeared to participate in the signaling pathway downstream of TLR3 and TLR7. Furthermore, another group demonstrated that an ssRNA fragment of SARS-CoV-2 genomic RNA was responsible for the activation of the TLR7/8-dependent MyD88 pathway in human DCs [45]. As TLR7/8 is activated by guanosine (G)- and uridine (U)-rich ssRNA [72], this group scanned putative TLR7/8 ligands within the SARS-CoV-2 genome and selected two GU-rich ssRNA sequences—termed SCV2-RNA—to test their hypothesis. Using human monocyte-derived DCs (MoDCs), the authors found that SCV2-RNA treatment induced the expression of pro-inflammatory cytokines, including TNF-α, IL-6, and IL-12, as well as the secretion of the T cell-recruiting chemokine CXCL9. SCV2-RNA-stimulated MoDCs exhibited a mature form and triggered cocultured CD4 and CD8 T cells to produce IFN-γ, suggesting that SCV2-RNA can mediate DC activation. Similar results were observed in pDCs. SCV2-RNA induced the upregulation of CD86 expression and the production of IFN-α and TNF-α in pDCs. The authors suggested that such ssRNA-induced activation is mediated via the TLR8/MyD88/NF-κB pathway in MoDCs and by TLR7 in pDCs. Taken together, these results indicate that ssRNA and dsRNA produced by SARS-CoV-2 are recognized by endosomal RNA sensors.

TLR9 recognizes CpG-rich DNA fragments derived from bacteria, viruses, and mitochondrial DNA (mtDNA) [60]. While the relationship between TLR9 and the recognition of SARS-CoV-2 infection is not clear, the coding region of the E protein and ORF10 in the SARS-CoV-2 genome was shown to be enriched with CpG [73]. Recently, TLR9 was suspected of inducing severe COVID-19 [42]. This TLR9-COVID-19 hypothesis, presented by Bezemer and Garssen, proposes that CpG islands in SARS-CoV-2 or mtDNA released from damaged host cells could trigger TLR9 activation and subsequently elicit pathogenic hyperinflammatory responses. As it is still unclear whether TLR9 directly detects viral components derived from SARS-CoV-2, future studies are needed to address this issue.

While it is well known that intracellular TLRs are physically separated from the cell surface to prevent autoimmune responses following receptor activation by host nucleic acids [74], interestingly, several studies have suggested the cell surface positioning of endosomal TLRs in certain cell types [75,76,77,78]. Cell surface expression of TLR3 was observed in human fibroblast cell lines [79], mouse splenic CD8^+^ dendritic cells, and marginal zone B cells [80]. Mouse bone marrow (BM)-derived macrophages, BM-conventional dendritic cells, BM-plasmacytoid DCs and B cells expressed TLR7 on the cell surface [77]. TLR9 was expressed on the surface of mouse splenic DCs [78], monocytes [81], and human neutrophils [82]. These findings suggest that both the cell surface and intracellular expression of nucleic acid-sensing TLRs may participate in the activation of immune responses. Interestingly, human airway epithelial cells expressed TLR3, TLR7 and TLR9 on the apical cell membrane [76], indicating that nucleic acid-sensing TLRs on the epithelial cell surface may play a crucial role in innate responses against inhaled pathogens such as SARS-CoV-2. However, further studies are required to prove this hypothesis.

## 4. Conclusions

TLRs participate in the first line of defense against invading pathogens. They recognize a broad range of PAMPs derived from microorganisms, and the main function of TLRs is the ability to activate innate immune responses, including cytokine production. At present, TLR2, TLR4, TLR3, TLR7, TLR8, and TLR9 have been suggested as possible receptors capable of recognizing SARS-CoV-2 infection. Viral components including the S protein, E protein, ssRNA, and dsRNA seem to serve as ligands for these TLRs. TLR-mediated immune responses are essential for host protection; however, uncontrolled and exacerbated inflammation can result in pathologic responses such as tissue damage. In patients with COVID-19, robust innate immune responses and hyperinflammation were observed in severe cases [3,4,7], resulting in septic shock [83], acute lung injury [84], and acute respiratory distress syndrome [85]. Therefore, immunomodulatory therapeutic strategies, including the use of anti-inflammatory drugs, have been suggested as promising treatments for severe COVID-19. Many approaches targeting TLR signaling pathways have been tested for their ability to attenuate hyperinflammation following SARS-CoV-2 infection. For example, the U.S. FDA has approved an investigation into the efficacy of a PUL-042 inhalation solution that blocks TLR2/6/9 to reduce the infection rate, progression, and disease severity of COVID-19 (NCT04312997, NCT04313023) [42,86]. Additionally, a clinical study to evaluate the inhibition of TLR3-mediated inflammatory responses by Famotidine [87] to improve outcomes in patients with COVID-19 has been conducted (NCT04389567, NCT04504240, NCT04724720, NCT04370262) [88,89]. Various TLR4 modulators, such as EB05 (NCT04401475), Eritoran (NCT02735707), Naltrexone [90] (NCT04604704, NCT04604678), Curcumin [91] (NCT04382040), and Berberine [92,93,94] (NCT04479202) are undergoing clinical trials for COVID-19. Although current studies have not produced sufficient encouraging results, based on the evidence presented in this review, targeting TLRs could be an effective treatment for COVID-19.

## Figures and Tables

**Table 1:** Toll-like receptors (TLRs) in humans.

TLRs	Ligands	Primary Localization	Adaptor Molecules	Signaling Characteristics	Refs.
TLR1	Triacyl lipopeptides	Cell surface	MyD88	Heterodimerization with TLR2	[46]
TLR2	Lipoproteins, Zymosan, etc	Cell surface	MyD88		[47,48]
TLR3	dsRNA	Intracellular	TRIF		[49]
TLR4	LPS, Viral envelope glycoproteins, etc	Cell surface	MyD88/TRIF		[50,51,52,53]
TLR5	Flagellin	Cell surface	MyD88		[54,55]
TLR6	Diacyl lipopeptides	Cell surface	MyD88	Heterodimerization with TLR2	[48]
TLR7	ssRNA	Intracellular	MyD88		[56,57]
TLR8	ssRNA	Intracellular	MyD88		[58]
TLR9	Unmethylated CpG-rich DNA fragment, mtDNA	Intracellular	MyD88		[59,60]
TLR10	Undefined	Cell surface	MyD88		[61,62,63]

## Data Availability

No new data were generated.
